# Electric-field control of nonlinear THz spintronic emitters

**DOI:** 10.1038/s41467-022-31789-0

**Published:** 2022-07-14

**Authors:** Piyush Agarwal, Lisen Huang, Sze Ter Lim, Ranjan Singh

**Affiliations:** 1 Division of Physics and Applied Physics, School of Physical and Mathematical Sciences, Nanyang Technological University, 21 Nanyang Link, Singapore, 637371 Singapore; 2 Center for Disruptive Photonic Technologies, The Photonics Institute, Nanyang Technological University, Singapore, 639798 Singapore; 3 Institute of Materials Research and Engineering A*STAR (Agency for Science, Technology and Research) 2 Fusionopolis Way, Innovis, Singapore, 138364 Singapore

**Keywords:** Ultrafast photonics, Terahertz optics, Magnetic devices, Electronic and spintronic devices, Electrical and electronic engineering

## Abstract

Energy-efficient spintronic technology holds tremendous potential for the design of next-generation processors to operate at terahertz frequencies. Femtosecond photoexcitation of spintronic materials generates sub-picosecond spin currents and emission of terahertz radiation with broad bandwidth. However, terahertz spintronic emitters lack an active material platform for electric-field control. Here, we demonstrate a nonlinear electric-field control of terahertz spin current-based emitters using a single crystal piezoelectric Pb(Mg_1/3_Nb_2/3_)O_3_–PbTiO_3_ (PMN–PT) that endows artificial magnetoelectric coupling onto a spintronic terahertz emitter and provides 270% modulation of the terahertz field at remnant magnetization. The nonlinear electric-field control of the spins occurs due to the strain-induced change in magnetic energy of the ferromagnet thin-film. Results also reveal a robust and repeatable switching of the phase of the terahertz spin current. Electric-field control of terahertz spintronic emitters with multiferroics and strain engineering offers opportunities for the on-chip realization of tunable energy-efficient spintronic-photonic integrated platforms.

## Abstract (web summary)

Spintronic terahertz (THz) emitters are a class of magnetic heterostructure where femtosecond laser excitations generate THz radiation emission. While they have great potential, electric field control of spintronic emitter remains a challenge. Here, by combining a spintronic emitter with a piezoelectric substrate, Agarwal et al. demonstrate electric field control of THz emission through induced piezostrain.

## Introduction

The terahertz band (0.1–30 THz) offers data rates scaling up to terabits per second^1,2^, surpassing the present state of the art. This makes THz devices prominent candidates for next-generation wireless communication and ultrahigh data processors^3–6^, providing a fundamental platform for scientific innovations^7,8^. Nonetheless, the devices continue to face major hurdles due to the lack of materials that could operate beyond existing electronic frequencies^9^. The recent discovery of the THz spin currents at sub-picosecond timescale^10–14^ has offered a path of considerable potential, enabling ultrafast data processing^15–19^ by femtosecond laser excitation of ferromagnets^20^. However, advanced device integration and applications^21,22^ require electrical control of the THz spin currents, preferably with a low-energy electric field.

Multiferroic materials have emerged as a strong candidate for achieving magnetoelectric effect at high speed and reliability^23,24^. Despite this, practical applications of single-phase multiferroic is limited due to the small magnetoelectric effect at room temperature^25^, and in lieu, synthetic multiferroic materials composed of ferroelectric (FE)/ ferromagnetic (FM) heterostructure provides an efficient path forward^26,27^. Owing to its intrinsic property, several device configurations have highlighted electrical-write magnetic-read applications^28–30^ by utilizing long-range strain-mediated coupling across the interface of FE and FM layers^31^, albeit limited to gigahertz frequencies^32,33^.

Here, we demonstrate a class of electric-field tunable nonlinear THz spintronic devices based on synthetic multiferroic materials. We engineer the electric-field induced strain^34,35^ of the PMNPT [011] substrate, *(1–x)*Pb(Mg_1/3_Nb_2/3_)O_3_–*x*PbTiO_3_; *x* = 0.31^36,37^ and control the spin distribution across the magnetic anisotropy barrier in the FM layer^38^, which governs the polarized spin current in the FM layer^39,40^. As a consequence, femtosecond photoexcitation of FM generates tailored ultrafast superdiffusive spin transport^41–43^. This transient spin current $({{{{\bf{j}}}}_{{{\bf{s}}}}})$ superdiffuse into an adjacent heavy-metal layer where it experiences inverse spin Hall effect (ISHE) to form a transverse charge current $({{{{\bf{j}}}}_{{{\bf{c}}}}})$ acceleration, leading to emission of terahertz radiation^11,44,45^. The essential feature of our demonstration includes: (i) observation of a nonlinear butterfly loop behavior of THz pulse amplitude upon cyclic control of the electric field at the remnant magnetic field. The curve follows the electric-field tuning of strain in PMNPT^46–48^, which we describe as THz-E hysteresis and show an electric-field controlled THz emission with a large modulation of upto 270% at remnant magnetization. (ii) A robust and repeatable electric-field switching of the phase of emitted THz pulse. (iii) Impact of magnetic anisotropy in THz emission is explored, and (iv) effect of varying PMNPT substrate thickness and laser fluence further reveals a passive and an active route to optimize the coercive electric field (*±E*_*cr*_) of the ferroelectric system for energy-efficient tailoring of THz pulse emission. All of these features provides an approach to design electric-field controllable nonlinear THz spintronic emitter device, although a recent work has shown amplitude modulation, albeit with a degrading performance over multiple modulation cycles^40^.

## Results

**Fig. 1:**
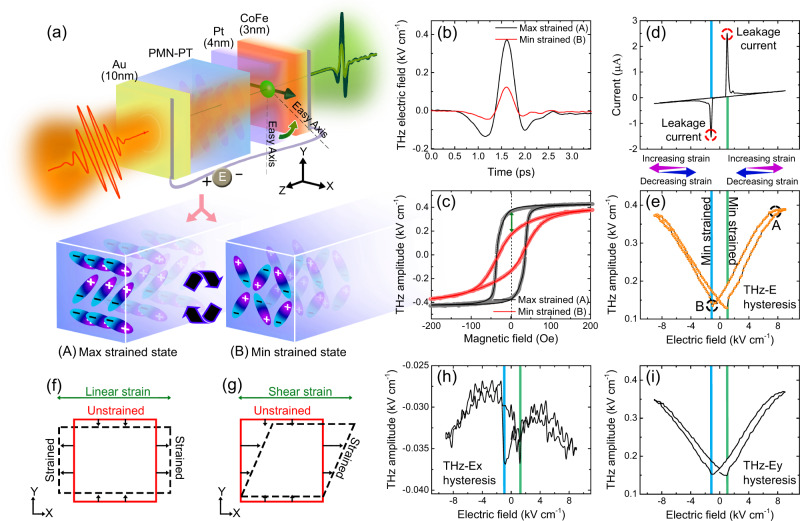
Electric field tunable THz spintronic emitter. **a** Image depicts the design of THz spintronic emitter, as used for the experiment, Laser beam (0.4 mJ cm^-2^)/ Au(10 nm)/ PMNPT(1 mm)/ Pt(4 nm)/ Co_75_Fe_25_(3 nm). PMNPT(011) is used as the ferroelectric material where the electric dipoles align in the (i) out-of-plane direction under maximum strained state and (ii) in-plane direction under minimum strained state. Upon strain transfer from the FE layer, the easy axis of the FM layer rotates as shown by the green arrow. **b** THz pulse as obtained from the emitter under two extreme strained states, A (max) and B (min) at *H* = *0* *Oe*. **c** THz-H hysteresis^51^ curves of the emitter under two extreme strained states. The green arrow highlights the observed change in the THz spin current at *H* = *0* *Oe*. The double Langevin model^61^ was used to fit the obtained THz-H hysteresis. **d** I-V characteristic shows the flow of leakage current from the substrate when sweeping the electric field between 9 kV cm^−1^ and −9 kV cm^−1^ at *H* = *0* *Oe*. The position of leakage current identifies the electric field coercivity in PMNPT, *E*_*cr*_ = ±1.2 kV cm^−1^. The blue line depicts negative *E*_*cr*_, and the green line depicts positive *E*_*cr*_ **e** THz-E hysteresis curves as observed upon recording the THz pulse amplitude at *H* = *0* *Oe* while sweeping the electric field between 9 kV cm^−1^ to −9 kV cm^−1^. **f**, **g** illustration of the two possible states of strain in the PMNPT as a result of applied electric field. **f** Linear strain **g** Shear strain **h** X-component of the THz-E hysteresis upon sweeping the electric field between 9 kV cm^−1^ to −9 kV cm^−1^ at *H* = *0* *Oe* **i** Y-component of the THz-E hysteresis upon sweeping the electric field between 9 kV cm^−1^ to −9 kV cm^−1^ at *H* = *0* *Oe*.

To investigate the magnetoelectric effect (ME) for tailoring the THz spin current, we fabricate a heterostructure of Pt(4 nm)/CoFe(3 nm) over a 1 mm thick substrate of PMNPT(011), as shown in Fig. 1a. A 10 nm thick layer of Au is deposited on the flip side of the substrate to create a uniform electrode across the thickness along the *z*-axis. Refer to the Methods section for detailed information on sample fabrication. Here, upon application of the electric field, the substrate attains a strained state where the electric dipoles within the PMNPT orient in the out-of-plane direction exerting a compressive strain on the FM layer.  Once the strain is removed, the dipoles re-orient in the in-plane direction of the substrate, releasing the strain from the FM. A further small antiparallel electric field (*±E*_*cr*_) is utilized to get rid of electric field retentivity from the PMNPT and drive the system to a minimum strain state. Refer to Supplementary Section S1 for a detailed explanation of the polarization control in PMNPT.

The magnetoelectric (ME) effect in PMNPT gives rise to both strain and charge-mediated coupling between the FE and FM overlayers^49^. Together, this allows for an enhanced ME effect but distinguishing between the two co-existing effects is challenging. Therefore, we suppressed the charge-mediated coupling by sandwiching a heavy metal (HM) layer between the FE and FM layers, which also acts as a layer causing inverse spin Hall effect converting spin current into charge current. This ultrafast spin to charge conversion in the HM layer leads to THz emission where the pulse amplitude provides a direct estimation of THz spin current^50^ $\left({{\bf{j}}}_{{{\bf{s}}}}\right)$, ${{{{{\bf{E}}}}}}_{({{{{{\rm{t}}}}}})}^{{{{{\bf{peak}}}}}}\,\propto {{{{{\bf{j}}}}}}_{{{{{\bf{c}}}}}}^{{{{{\bf{peak}}}}}}$ where **j**_**c**_ = γ**j**_**s**_ × **M**/|**M**|^45^, **(γ)** is the spin Hall angle of the HM layer and **M**/|**M**| denotes the FM magnetization unit vector. Figure 1b demonstrates the terahertz electric field measured at the two extreme strained electric field states with a THz peak modulation of 270% at the remnant magnetic field (refer to Supplementary Section S5 for THz pulse at all the electric fields). A measured THz amplitude with a cyclic magnetic sweep (THz-H hysteresis^51^, refer to methods section for experimental details) is shown in Fig. 1c, and indicates a substantial change in the magnetic anisotropy by applying an electric field. Such release of strain in PMNPT is caused through the generation of leakage current where the dipoles lose the polarization due to random alignment throughout the substrate. A detailed investigation of electric field-induced strain in the PMNPT substrate is also performed using the impedance matching method^52^ (refer to Supplementary Section S8 for details). A strain ranging from 0.053% to −0.008% (or 0.53 to −0.08 µm) is induced in the 1 mm substrate with the application of an electric field in the range of ±6 kV cm^−1^. Ferromagnet/heavy metal heterostructure deposited over the PMNPT substrate thus experiences the same degree of strain. Concurrent with the leakage current generation, as shown in Fig. 1d, the substrate exhibits a rapid decrease in strain. In particular, at these instants, arising at *E*_*cr*_ *=* ±1.2 kV cm^−1^, the system attains a minimum strained condition, as shown in Fig. 1d. When the THz pulse amplitude (at *H=0* *Oe*) is recorded across the electric field, a remarkable similarity with the known butterfly loop^46–48^ of the strain is observed, with minimum amplitude at *±E*_*cr*_. We henceforth term this hysteresis as *THz-E butterfly hysteresis* (refer to methods section for experimental details). The overall strain-induced effect can be explained by the rotation of easy axis of the FM under the effect of linear strain (Fig. 1f); however, recent studies have also revealed a possible rotation of FM magnetization caused by the shear strain^31,53,54^ (Fig. 1g). To distinguish, we investigated the *x* and *y* component of THz-E hysteresis. Typically, the THz emission is polarized perpendicular to the FM magnetization; therefore, in the presence of any magnetization rotation, a complementary change in the *x* and *y* component of THz-E hysteresis is expected. However, Fig. 1h and i shows no such significant change in the THz-E_*x*_ hysteresis and no complementary signature in THz-E_*y*_ hysteresis. This provides evidence for a constant polarization of the emitted THz pulse and suggests the presence of easy axis rotation of the FM under the applied electric field. The rotation of the easy axis can be further described approximately with the remnant magnetization ∝ |cos (φ_H_−φ_easy_)|, where φ_easy_ is the uniaxial easy axis direction and φ_H_ as the applied magnetic field direction^55^. Since the THz-E hysteresis follows the remnant magnetization, we therefore relate the THz amplitude ∝ |cos (φ_H_−φ_easy_) | and calculate the easy axis rotation angle given by φ_H_−φ_easy_. As shown in Supplementary Section S7, the results demonstrate close to 90-degree rotation in the easy axis, thus aligning it along the hard axis and vice-versa.

**Fig. 2:**
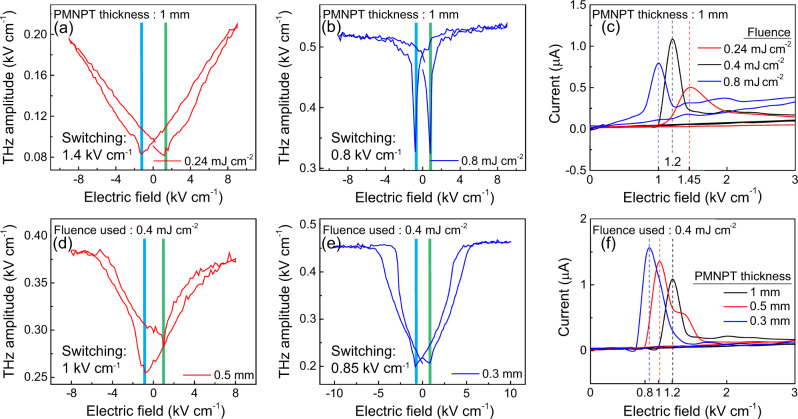
Electric field control of emitted THz amplitude with varying laser fluence and PMNPT thicknesses, at *H=0 Oe*. **a**, **b** THz-E hysteresis recorded with PMNPT thickness of 1 mm at a laser fluence of **a** 0.24 mJ cm^−2^ **b** 0.8 mJ cm^−2^ **c** I-V characteristic highlights the quenching in electric field coercivity with increasing laser fluences as seen through decreasing peak positions **d**, **e** THz-E hysteresis recorded at an applied laser fluence of 0.4 mJ cm^−2^ with PMNPT thickness of **d** 0.5 mm **e** 0.3 mm. **f** Decrease in the leakage current peak positions with decreasing PMNPT thicknesses shows quenching in electric field coercivity. The I-V characteristic of the emitter with 1 mm thick PMNPT substrate and pump fluence of 0.4 mJ cm^−2^ is adapted from Fig. 1(d).

We further investigate the electric-field dependence of strain and optimize the devices.  First, the laser fluence was varied for the device illumination. It was earlier demonstrated that the coercivity of the FM can be altered by photo-thermal effect^51,56^ through heating of the substrate. Following a similar approach, we recorded a THz-E hysteresis loop at two other fluences, 0.24 and 0.8 mJ cm^−2^_,_ alongside the previous THz-E hysteresis loop at 0.4 mJ cm^−2^, as shown in Fig. 1e. In Fig. 2a and b, we notice that lower fluence exhibits similar behavior as in Fig. 1e, whereas a contrasting behavior is observed for a higher fluence of 0.8 mJ cm^−2^. Moreover, the THz amplitude increases with higher fluence due to enhanced spin excitation. The electric field coercivity is observed to change with the laser fluence and thus opens an active route for tuning the nonlinear control, as shown in Fig. 2c. Next, a passive control of THz-E hysteresis is explored by varying the substrate thickness in Fig. 2d and e. As shown in Fig. 2f, we observe a similar control over the electric field coercivity as was observed for active control of fluence (Fig. 2c). Besides, when converted to the applied voltage (based on the substrate thickness), a more significant decrease in the switching voltage is realized, displaying a route for scaling down the applied voltage. PMNPT operating at room temperatures offers large device scalability down to micron size, making the platform energy efficient for memory applications. Various recent studies have focused on demonstrating a high repetition rate revealed by a smaller array of microstrips. One such example is a recent work by Slawomir et al.^33^, where they demonstrated strain by applying 1 kV cm^−1^ with speeds of up to 100 MHz using micron-size thin strips of ferromagnet/PMNPT. However, here we used a thin-film-based THz spintronic emitter with a much larger dimension that rendered the device unsuitable for fast modulation speed (Refer to Supplementary Section S10 for modulation experiments of our device).

**Fig. 3:**
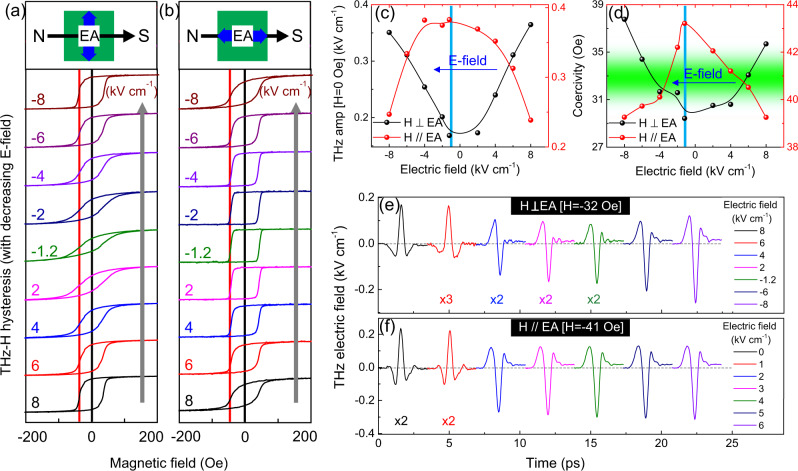
Electric field controlled phase reversal of THz spin current. **a**, **b** Gradual evolution of THz-H hysteresis in the device when electric field is swept from 8 kV cm^−1^ to −8 kV cm^−1^ at pump fluence 0.4 mJ cm^−2^. The solid black line is depicted at the remnant magnetic field, and the solid red line is depicted at the switching magnetic field. The results are observed when the applied magnetic field is **a** perpendicular to the easy axis of the FM layer, **b** parallel to the easy axis of the FM layer. **c** One-to-one comparison of the remnant magnetization of the FM layer for H⊥ EA (Black balls and solid black line) and H// EA (Red balls and solid red line). **d** One-to-one comparison of the coercivity of the FM layer for H⊥ EA (Black spheres and solid black line) and H// EA (Red spheres and solid red line). The area highlighted in the green shows the region of interest for accessing the THz phase reversal. **e**, **f** Sequential change in the THz phase upon the applied electric field on the device when **e** H⊥ EA; H= −32 Oe **f** H// EA; H = −41 Oe. **e**, **f** The pulses are shifted in the *x*-axis to show the applied electric-field dependent phase reversal of the emitted THz pulses.

Another important goal is to realize a phase reversal of the spin current that can establish the practical application for terahertz spintronic devices. We investigate this feature in two separate configurations of the devices, wherein the applied magnetic field is (i) perpendicular to the easy axis (EA) of FM and (ii) parallel to the easy axis (EA) of FM. In Fig. 3a and b, we represent the gradual evolution of the THz-H hysteresis for the two configurations while the electric field is swept from 8 to −8 kV cm^−1^. Note that earlier measurements shown in Figs. 1 and 2 were performed with a magnetic field oriented perpendicular to the easy axis, as shown in Fig. 3a. However, a striking difference is observed between Fig. 3a and b. In Fig. 3a, we notice a square-like THz-H hysteresis loop for strained PMNPT and a non-square-like loop for its unstrained counterpart. In contrast, for Fig. 3b, the strained PMNPT exhibits a non-square like THz-H loop and a square-like behavior in the unstrained case. These differences can be understood as easy axis rotation^57^, where, upon application of electric field, H⊥EA tends to H//EA, and H// EA tends to H⊥EA. Following that, Fig. 3c and d plots the relative change in the remnant magnetization and coercivity of the FM layer. Here, we observe a complementary behavior in the two cases with the same *E*_*cr*_ *=* ±1.2 kV cm^−1^. Further, the change in the coercivity, shown in Fig. 3d, opens the route to an important aspect of the electric-field control. This reveal a region of interest where, if a device is configured at a magnetic field just below its coercivity, as shown by the green region, an applied electric field can switch the spin state, as observed through the THz phase reversal in Fig. 3e and f for H⊥ EA and H// EA, respectively.

**Fig. 4:**
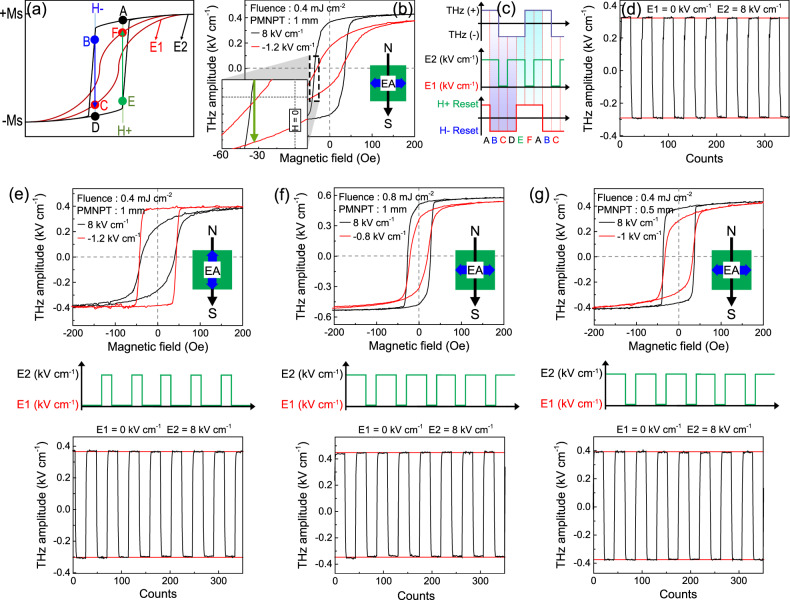
Electric field controlled THz phase switching for practical devices. **a** Figure illustrating the two hysteresis curves at an applied electric field of E1 (low strained state, solid red line) and E2 (maximum strained state, solid black line). The reduction in coercivity opens the route to switch the THz phase using electrical field along the path B to C and E to F at magnetic fields H- and H + , respectively. **b** THz-H hysteresis at two extreme electric field states, 8 kV cm^−1^ and −1.2 kV cm^−1^ for H⊥ EA, fluence = 0.4 mJ cm^−2^ and PMNPT thickness =1 mm. **c** An illustrative figure to depict a route to electric field control of the THz phase using the two magnetic reset states, H + Reset and H- Reset (shown in a red curve; bottom) The electric field control is shown in green curve (middle), and the THz phase is shown in black curve (top). **d** THz endurance demonstrated on the device with twenty sets of data at each extreme states highlighting a robust and repeatable operation. **e**, **f**, **g** Set of THz-H hysteresis, applied electric field, and THz endurance for the case where either **e** applied magnetic field direction is changed to H// EA or **f** fluence is enhanced to 0.8 mJ cm^−2^ or **g** PMNPT substrate thickness is decreased to 0.5 mm.

Figure 4a better illustrates the above phenomena by using two hysteresis curves at different applied electric field strengths, *E1* and *E2*, to achieve a minimum and maximum strain state, respectively. It can be observed that an applied magnetic field right below the coercivity enables the electric field to drive the device from point B to point C through spin polarization reversal. Such behavior is due to additional energy derived from the electric-field that steers the spins across the magnetic anisotropy barrier leading to electrical control of the THz phase. A similar phase reversal in the opposite direction can be realized from E to F with a positive applied magnetic field. Figure 4b highlights this use of coercive field change where, according to Fig. 3d, the switching magnetic field is chosen to be −32 Oe. Notice that the amplitude for THz spin current appears attenuated during the THz phase reversal; however, it can be enhanced. For example, if a device experiences a phase reversal through attenuated states in points B and C, the spin-current can be again enhanced following the path from C to D (negative THz phase with higher amplitude). Here, the applied magnetic field acts as a reset switch, as shown in Fig. 4c. For an H- Reset state [during the B-C-D path], it can be observed that the *E2* electric field initially yield a positive phase THz spin current, but upon switching the field to *E1*, the THz phase reverses to negative sign [although with attenuated amplitude]. However, after resuming at *E2*, the spin-current continues to stay in the negative phase [with enhanced amplitude]. The demonstration highlights the potential to control the phase of THz spin current using an electric field and additional magnetic reset control. Figure 4d demonstrates this result on our basic device configuration with H⊥ EA, using *E1=*0 kV cm^−1^ and *E2* *=* 8 kV cm^−1^. Twenty sets of data points recorded in both [C-D-E] and [F-A-B] states for a total of 350 counts with high reliability and repeatability was observed for an electric field controlled phase of THz spin current. Note that, in the earlier case, *E2* electric field was applied with an intermediate switch-off to change the phase of the THz spin current. However, for examining a similar THz phase reversal when H// EA (Fig. 4e), only a trigger switch-on of the electric field is required, which can otherwise remain switched off at all other times. Figure 4e thus exhibits the complementary control depicted over the THz-H hysteresis and a robust endurance pattern for similar phase control with higher energy efficiency. Figure 4f further demonstrates the robust endurance for device application at higher fluence by utilizing a lower *E*_*cr*_. Figure 4g also demonstrates a similar application in a thinner substrate where the switching E-field voltage can be reduced. Such potential applications in magnetic memory may surpass current technologies due to their inherent non-volatile feature, higher frequency operation, and low power consumption^58^. On an elementary scale, magnetic memories are composed of an array of magnetic tunnel junctions where the writing process involves the transport of spin current but is limited in the frequency of operation^59^. Therefore, in this work, we reveal a pathway to take advantage of terahertz operation frequency arising from an ultrafast spin current generation at sub-picosecond timescales, demonstrating electric-field tunability with energy-efficient stimulus. To further investigate the electric-field controllable terahertz spintronic emitters for additional proof of concept, a comprehensive study was also performed on a different ferromagnet, CoFeB, as given in Supplementary Section S6.

In summary, this work presents electric-field control of nonlinear THz spintronic emitter, mediated through strain transfer across FE/FM interface in a synthetic multiferroic system consisting of PMNPT integrated with an FM/HM heterostructure. A unique investigation tool, THz-E butterfly hysteresis, is utilized to study and demonstrate the nonlinear strain effect imparted on the FM layer. The electric-field control enables 270% tuning of emitted THz amplitude. Reliable and repeatable control over the phase of THz spin current is demonstrated for its extensive application in device operation. Our work opens a direction for synthetic multiferroic-based electric-field controllable THz spintronic emitter with nonlinearities that would serve as an ideal next step for the development of on-chip dynamic terahertz spintronic-photonic devices.

## Methods

### Device fabrication

For investigation of the electric-field controlled THz spin current, PMNPT-0.31 (011) was used as a ferroelectric substrate. Overall, three different thicknesses of double-sided polished PMNPT substrate were investigated in this work, 0.3 mm, 0.5 mm, and 1 mm. Two different types of spintronic THz emitter were fabricated using ferromagnets, Co_75_Fe_25_ and Co_40_Fe_40_B_20_. 3 nm of ferromagnetic films and 4 nm of Platinum films were sputtered using the Singulus Timaris magnetron sputtering system with a vacuum base pressure of <5×10^−8^ mbar at a power of 0.5 kW. In addition, 10 nm Au was thermal deposited at 0.1 A/s with vacuum base pressure of <7×10^−6^ mbar at a power of 80 W. The substrates consist of the same batch, and thus same growing conditions were ensured. Electrode wires were attached on both sides of the sample using silver paste and cured over the hot plate for a half-hour each. I-V measurements confirmed the clean electrode deposition and wire connectivity. As shown in Supplementary Section S3, we have measured the M-H loop at different electric fields to ensure the butterfly loop. We did not observe any significant role of minor processes that could result in terahertz emission (Supplementary Section S4).

### Electric field calculation

The electric field indicated throughout the manuscript is calculated by the equation, $E-{field}=\frac{{{{{{\rm{Applied}}}}}}\,{{{{{\rm{voltage}}}}}}}{{{{{{\rm{Thickness}}}}}}\,{{{{{\rm{of}}}}}}\,{{{{{\rm{the}}}}}}\,{{{{{\rm{substrate}}}}}}}$. Note that for all the cases, the voltage is applied only across the thickness of the substrate.

### I-V measurements

Copper wires were used to make the electrode wiring on the sample, which connected to the source meter device, Keithley 2470. By use of a home-built LabVIEW code, the voltage was applied to the device, and the current flowing through the circuit was sensed.

### THz generation and measurement setup

Femtosecond laser pulses were used to generate the terahertz radiation from the spintronic emitter, which was detected using a 1 mm thick nonlinear ZnTe <110> cut crystal. Femtosecond laser pulses possessed the characteristic feature of 800 nm wavelength with a 1 kHz repetition rate and 35 fs pulse width. The parent laser pulse was split in a 90:10 ratio where the larger intensity laser beam was used as a pump to illuminate the device at a fluence of 0.4 mJ cm^−2^, well below the optical damage^60^. The lower intensity laser beam was used as a probe in a time-matched pump-probe configuration. The emitted THz pulse from the device was collected using the parabolic mirrors to focus onto the detector crystal to induce an optical birefringence. Due to this birefringence, the probe laser experiences a change in the polarization, proportional to the THz field strength. By use of the optical delay line in the path of the probe laser, the common time window was shifted to allow the probe laser to scan the THz electric field. For the electro-optic detection, a combination of a quarter waveplate and a Wollaston prism is used, which separates the *s* and *p* polarization of the laser. Rotation in the probe laser was then detected by a balanced photodiode which measures the difference in the intensities of these *s* and *p* polarized light. The detected electrical signal is first pre-amplified and then fed into the lock in to enhance the signal-to-noise ratio. The obtained signal from the lock in is used to construct the THz electric field using a home-built LabVIEW code. Refer to Supplementary Section S2 for a schematic diagram of the experimental setup. Refer to Supplementary Section S9 for a detailed calculation of the THz electric field.

### THz-H hysteresis measurement

For the measurement, the emitted terahertz pulse is monitored to find the peak THz delay position. Then, upon fixing the constant delay position corresponding to the terahertz pulse amplitude, the magnetic field of the sample is swept across the hysteresis H-field. This sweep of terahertz pulse amplitude with respect to the H-field yields a hysteresis curve called THz-H hysteresis. To ensure an accurate measurement, any possible eddy current in the electromagnet is avoided by using an acrylic housing for the magnetic coils. The core of the electromagnet is eliminated in the process to prevent any remnant magnetization in the measurement. Before performing any measurement, a corresponding magnetic field value for the applied voltage is measured and thus calibrated at the center of the electromagnet using a gaussmeter. After placing the sample at the same position, the voltage to magnetic field calibration is implemented to calculate the H-field applied to the sample. The gap between the electromagnetic coils is much more than the sample size of ~ 1 cm x1cm. A uniform magnetic field with fluctuation of ~0.1 Oe was observed in the system for the given sample space.

### THz-E butterfly hysteresis measurement

For the THz-E measurement, the delay position is fixed at the THz pulse amplitude position. Thereafter the applied electric field is swept through the sample, and the THz pulse amplitude is recorded simultaneously. To maintain uniformity, the electric field is first scaled from maximum to minimum value, and then to the zero-field, where the data recording starts. Here, the data is recorded up to the maximum field, then driven to the minimum field, and at last, brought up to zero electric field. In addition, the removal of magnetic energy is required in the data to prevent any convolution of the effect from both applied electric and magnetic fields. Therefore, to record the perfect change at the remnant magnetization, care is taken to switch on and off the maximum magnetic field before recording each point over the THz-E hysteresis measurement. In addition, THz-E_x_ and THz-E_y_ measurements were also recorded in this work. To perform these measurements, a THz wire grid polarizer is used in two orthogonal directions that filter out the desired component.

### THz endurance measurement

Terahertz endurance measurement is performed on the devices to realize the robustness and reliability of data operations over multiple cycles. In order to do this, the THz amplitude is recorded while the spintronic heterostructure is swept across the points A-F, by tailoring the applied electric and magnetic field as shown in Fig. 4c. Twenty sets of data points are recorded in both [C-D-E] and [F-A-B] states for a total of 350 counts.

## Supplementary information

Supplementary Information

## Data Availability

The authors declare that all data supporting the findings of this study used in this article and its supplementary information are openly available in NTU research data repository DR-NTU at 10.21979/N9/ZKGPGD. Additional information related to this paper is available from the corresponding author, R.S., upon reasonable request.
