# Supplementary material for: Electric-field control of nonlinear THz spintronic emitters
Source: Nat Commun. 2022 Jul 14;13:4072. doi: 10.1038/s41467-022-31789-0 (PMC9283400; doi:10.1038/s41467-022-31789-0)
Supplement: Supplementary file 1 — Supplementary Information [file 41467_2022_31789_MOESM1_ESM.pdf]

Supplementary Information

Electric-field control of nonlinear THz spintronic emitters

Piyush Agarwal, Lisen Huang, Sze Ter Lim, Ranjan Singh*

P. Agarwal, Prof. R. Singh
Division of Physics and Applied Physics, School of Physical and Mathematical Sciences, Nanyang Technological University, 21 Nanyang Link, Singapore 637371, Singapore
E-mail: ranjans@ntu.edu.sg

P. Agarwal, Prof. R. Singh
Center for Disruptive Photonic Technologies, The Photonics Institute, Nanyang Technological University, Singapore 639798, Singapore
E-mail: ranjans@ntu.edu.sg

Dr. L.S. Huang, Dr. S.T. Lim
Institute of Materials Research and Engineering A*STAR (Agency for Science, Technology and Research) 2 Fusionopolis Way, Innovis, 138364, Singapore

Keywords: electric-field controlled terahertz spintronic emission, strain-engineered spintronic heterostructures, THz-E hysteresis, phase and amplitude modulation, large endurance, synthetic multiferroics

Supplementary Section S1: Electric field controlled polarization switching in (011) oriented PMNPT

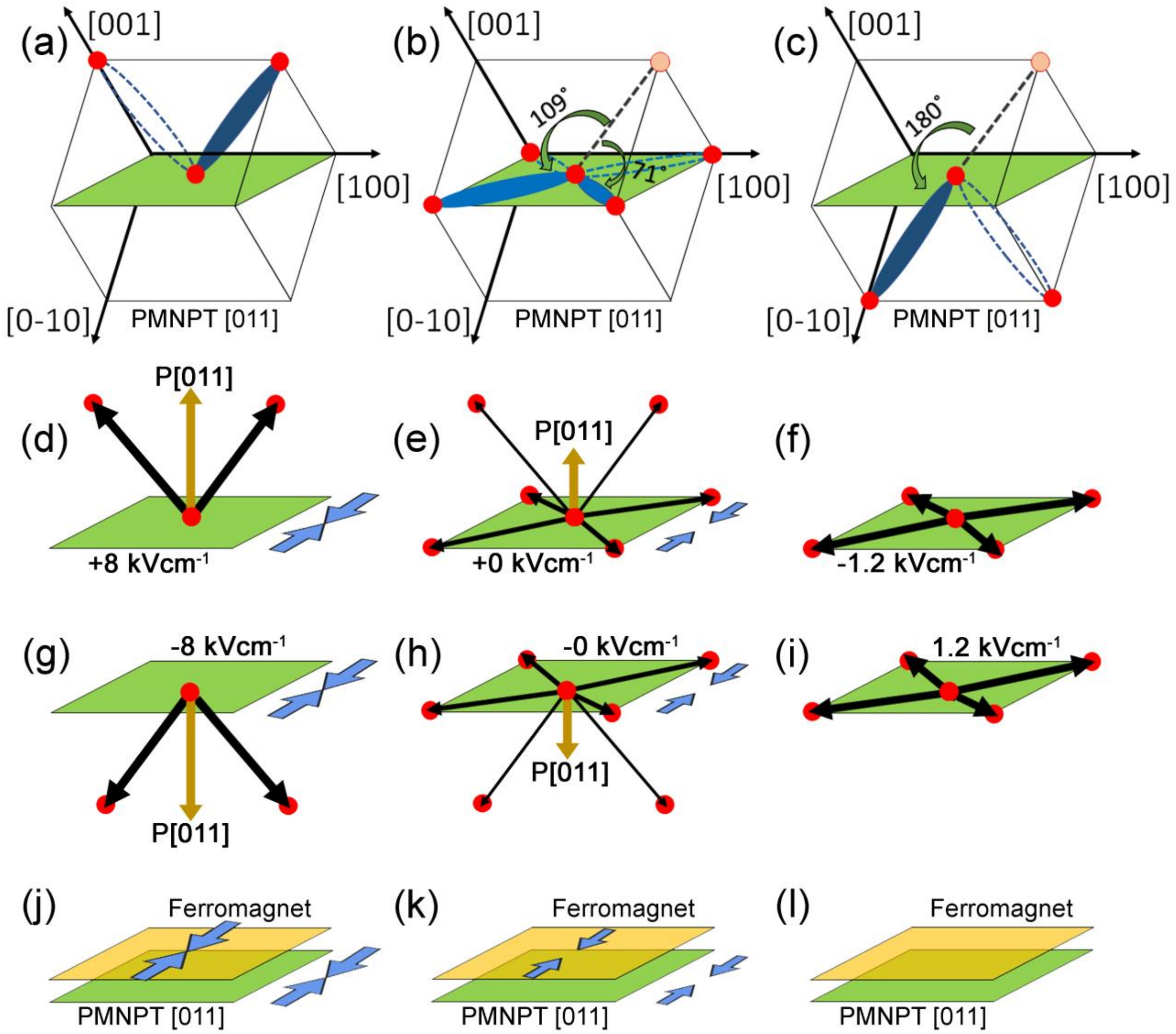

Supplementary figure S1: Electric field controlled dipole polarization in PMNPT(011). (a-c) figure illustrating a unit cell of PMNPT in its pseudo-cubic configuration. The electrical dipole in each unit cell is allowed to align in 8 different orientations, as shown in the shaded and dotted outline. (d-i) The resulting dipole orientation leads to a varying out-of-plane polarization based on

the applied electric field and manifest the strain in the substrate. (j-l) the ferromagnet, grown as an overlayer above PMNPT, experiences the same strain effect.

As shown in Supplementary figure S1(a-c), the electric dipole within PMNPT is allowed to align in either of the eight orientations. Here, upon application of the electric field, the dipoles prefer to orient in the out-of-plane direction and therefore choose either the (a) or (c) configuration. On the other side, when the substrate is in unpoled condition, the dipoles prefer to orient in the in-plane direction as shown in (b), and while transforming from poled to an unpoled case, it undergoes two possible non-180˚ rotations, 109˚ and 71˚.

Further, as shown in Supplementary figure S1(d-i), the dipoles rotate upon application of an electric field, and a net perpendicular polarization appears on the sample surface. Simultaneous to this polarization, a compressive strain is induced in PMNPT. Upon electric field reversal, the dipole polarization reverses likewise; however, the strain effect behaves symmetrically.

This strain effect extends to the entire area of the thin films grown on the top of the substrate. Therefore, as shown in Supplementary figure S1(j-l), the spintronic heterostructure experiences a resultant compressive strain and alters the magnetic anisotropy of the FM layer[1].

Supplementary Section S2: Spintronic terahertz setup

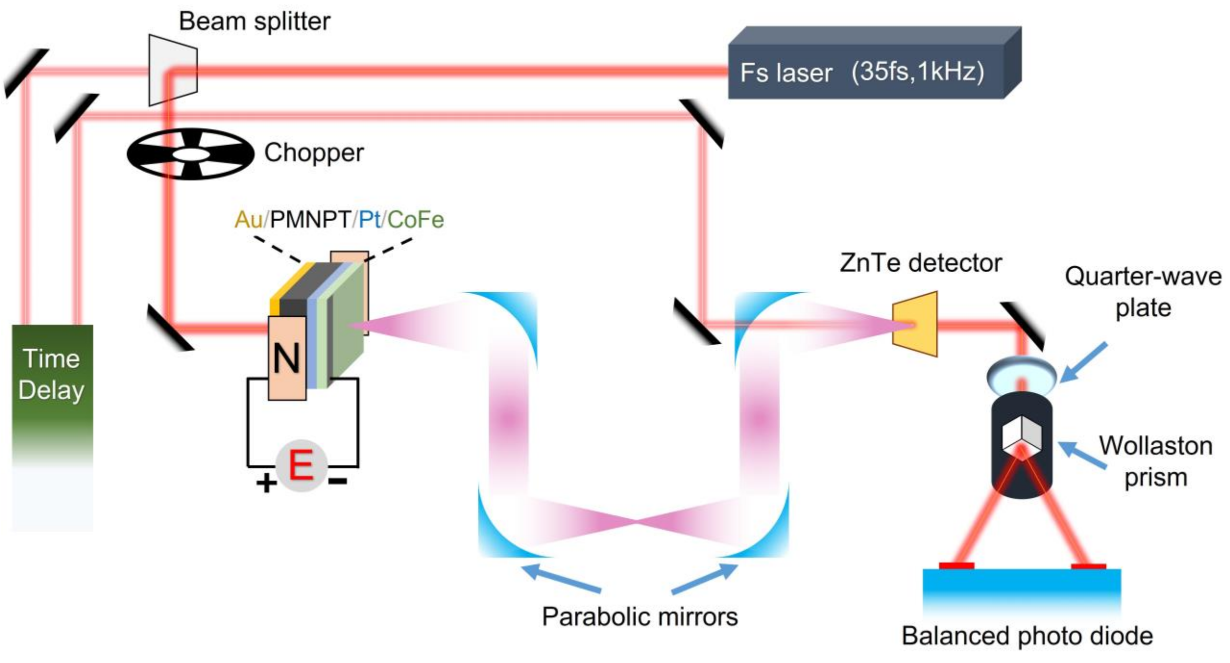

Supplementary figure S2: Schematic of the experimental setup used to generate and probe THz pulses in the presence of magnetic field and applied electric field.

Supplementary figure S2 shows the complete setup for THz pulse generation and detection used in the manuscript. The details of the experimental procedures are provided in the Methods section of the main manuscript.

Supplementary Section S3: Vibrating sample magnetometer (VSM) experiment

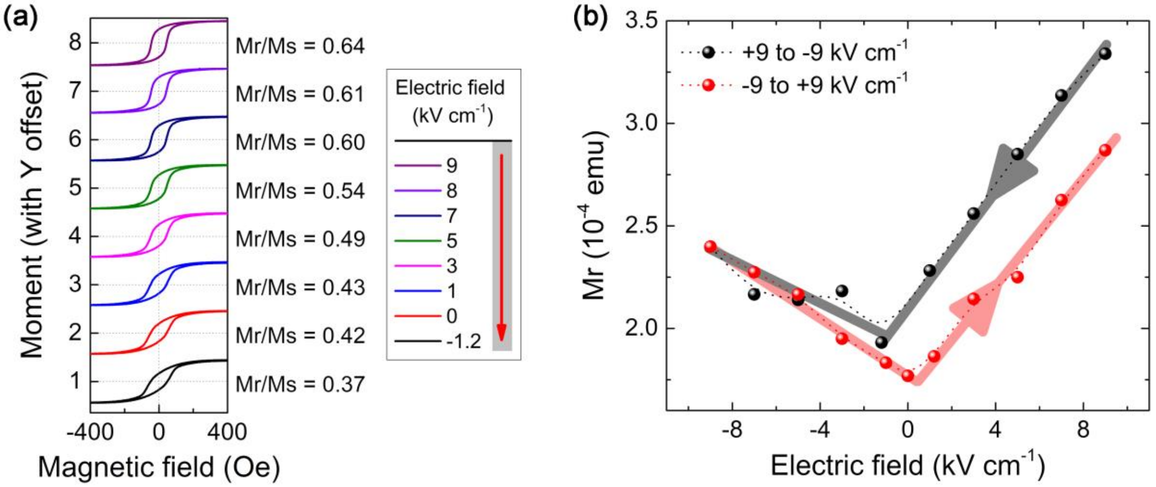

Supplementary figure S3: (a) M-H loop recorded for PMNPT/Pt(4nm)/CoFe(3nm) while sweeping the electric field from 9 kV cm^{-1} to -1.2 kV cm^{-1}. (b) Scaling of remnant magnetization observed in the emitter upon applied electric field between 9 kV cm^{-1} to -9 kV cm^{-1} (black balls with solid black line as a guide) and -9 kV cm^{-1} to 9 kV cm^{-1} (red balls with solid red line as a guide)

Supplementary figure S3 depicts a similar butterfly loop behavior as seen through the THz-E hysteresis curve. The switching voltage was observed at the same electric coercivity, E_{cr} = ±1.2 kV cm^{-1}. A partial difference as compared to THz-E hysteresis stems due to possible imperfections involved in the experiment.

Supplementary Section S4: Eliminating the role of minor processes in resulting terahertz emission

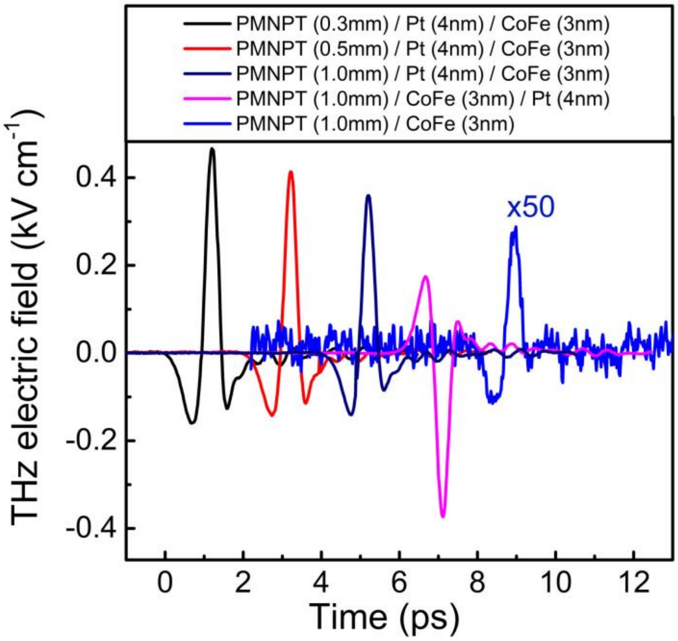

Supplementary figure S4: Control experiments to compare the terahertz emission from (i) PMNPT(0.3mm)/Pt/CoFe (solid black line) (ii) PMNPT(0.5mm)/Pt/CoFe (solid red line) (iii) PMNPT(1.0mm)/Pt/CoFe (solid navy line) (iv) PMNPT(1.0mm)/CoFe/Pt (solid pink line) and (v) PMNPT(1.0mm)/CoFe (solid blue line).

As shown in Supplementary figure S4, experiments were performed to estimate a relative THz emission from the samples used for the experiment [at *H=200 Oe*]. An additional check performed with Au/PMNPT(1.0)/CoFe reveals that there is no significant THz contribution arising out of the electric dipoles of PMNPT or Au electrode. A constant fluence of 0.4 mJ cm^{-2} was used to illuminate the sample. A gradual decrease in the THz emission with increasing PMNPT thickness (Supplementary figure S4 (i-iii)) is observed due to increasing fluence absorption in the PMNPT substrate itself.

Supplementary Section S5: Emitted THz pulse controlled by application of electric field on PMNPT(1.0mm)/Pt/CoFe [H ⊥ EA]

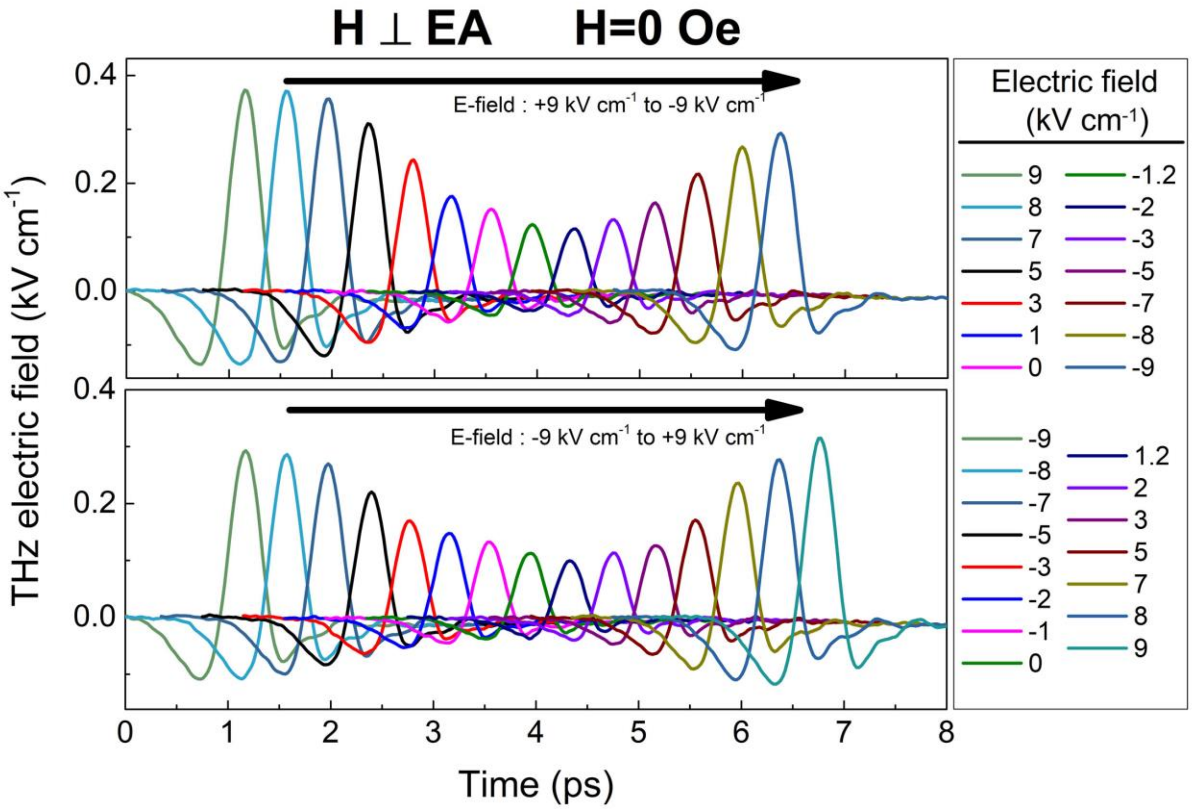

Supplementary figure S5: Detailed comparison of the THz pulses [at *H=0 Oe*] as it evolves upon application of electric field from +9 kV cm^{-1} to -9 kV cm^{-1} (top) and -9 kV cm^{-1} to +9 kV cm^{-1} (bottom). The sample was initially magnetized perpendicular to the easy axis. The pulses are shifted in the *x*-axis for demonstration of the change in THz amplitude.

Supplementary Section S6: Control experiments performed using the spintronic heterostructure with a different ferromagnet, CoFeB

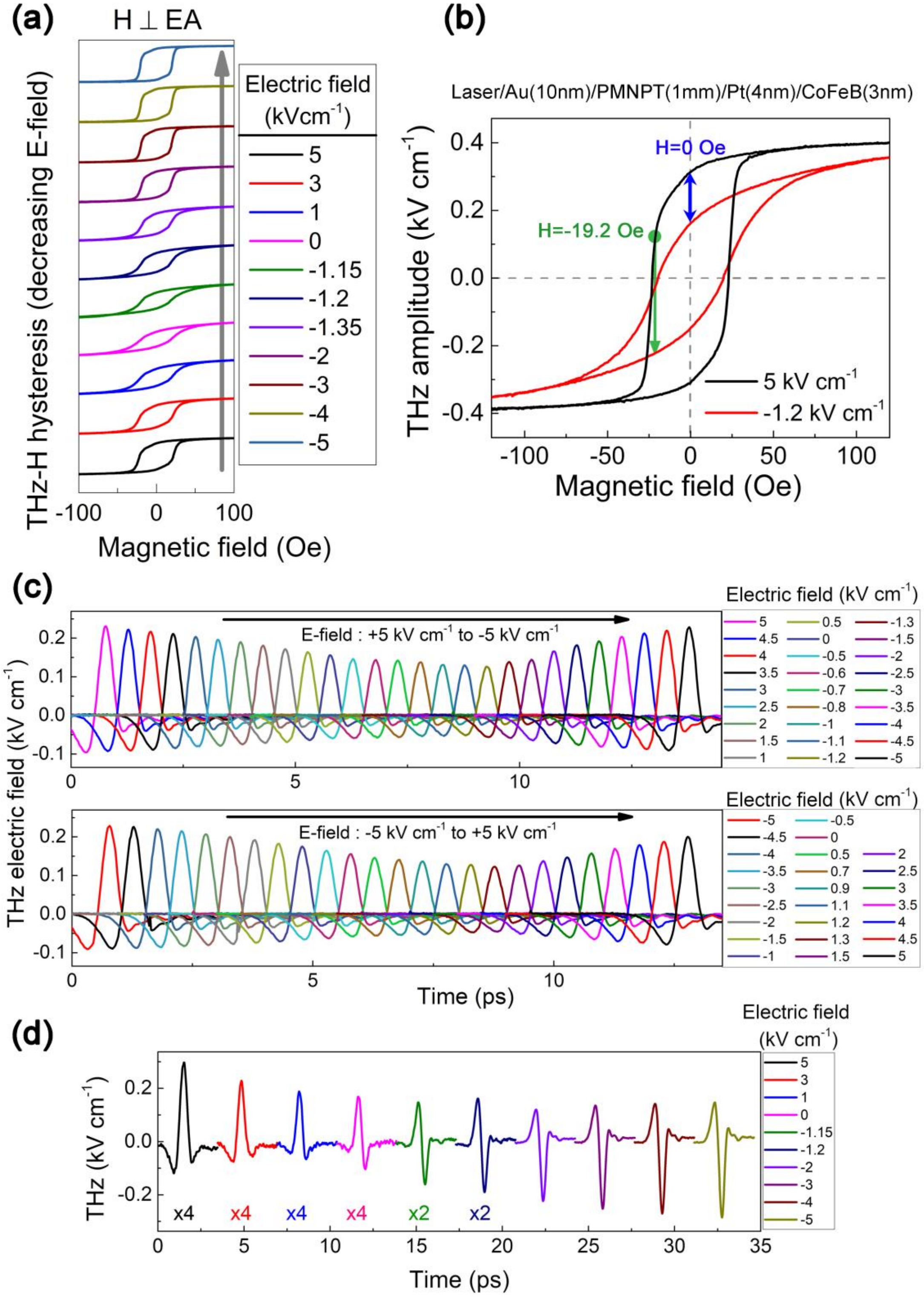

Supplementary figure S6: Control experiments performed using the spintronic heterostructure with a different ferromagnet, CoFeB, exhibiting (a) evolution of THz-H hysteresis while sweeping the electric field from 5 kV cm^{-1} to -5 kV cm^{-1}. (b) THz-H hysteresis at two extreme strained states highlights a decrease in coercivity which opens a prospective route to control the THz phase and amplitude through an electric field. (c) detailed comparison of the THz pulses [at *H=0 Oe*] upon application of electric field from +5 kV cm^{-1} and -5 kV cm^{-1} (top) and -5 kV cm^{-1} and +5 kV cm^{-1} (bottom). The pulses are shifted in the x-axis for easy demonstration of the change in THz amplitude. (d) detailed comparison of the THz pulses [at *H=-19.2 Oe*] as it evolves upon application of electric field from +5 kV cm^{-1} to -5 kV cm^{-1}. This change in the THz phase provides additional proof of concept to the main narrative of the manuscript. The pulses are shifted in the *x*-axis for easy demonstration of the change in the THz phase.

Supplementary Section S7: Quantification of easy axis rotation in PMNPT(1.0mm)/Pt/CoFe

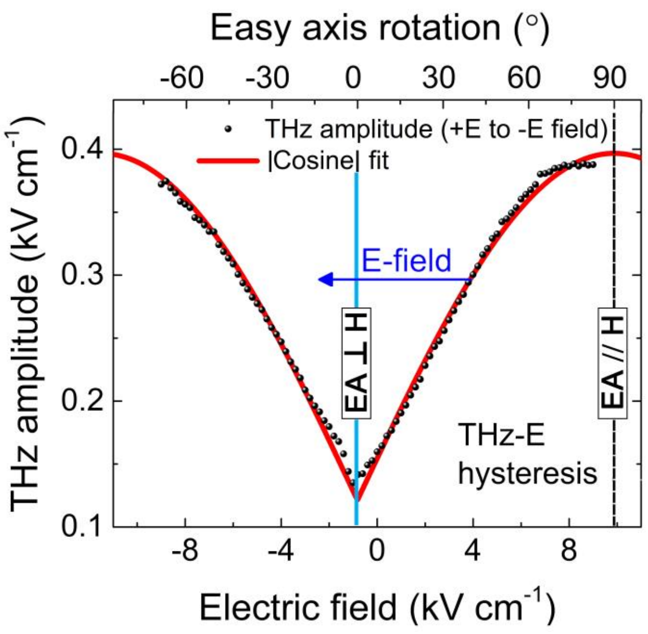

Supplementary figure S7: Quantification of easy axis rotation: THz-E hysteresis curve observed upon recording the THz pulse amplitude at *H=0 Oe* while sweeping the electric field from 9 kV cm^{-1} to -9 kV cm^{-1} in PMNPT(1.0mm)/Pt/CoFe. A solid red line shows a model cosine fit to find the approximate easy axis rotation in the sample upon application of the electric field. EA ⊥ H and EA // H states are indicated by the solid blue line and dashed black line, respectively.

The rotation of the easy axis can be described with the remnant magnetization $\propto |\cos(\varphi_H - \varphi_{easy})|$, where φ_{easy} is the uniaxial easy axis direction and φ_H is the applied magnetic field direction. Since the THz-E hysteresis follows the remnant magnetization, we, therefore, model the THz amplitude $\propto |\cos(\varphi_H - \varphi_{easy})|$ and derive the relative easy axis rotation[2] angle given by $\varphi_H - \varphi_{easy}$. The easy axis is demonstrated to rotate as high as ~90°, aligning it along the hard axis and vice-versa. The result is further justified through figures 3(a and b), where, with the application of electric field, an almost square shape of M-H hysteresis is achieved in both the cases of EA ⊥ H and EA // H.

Supplementary Section S8: Quantification of strain on the application of electric field over PMNPT

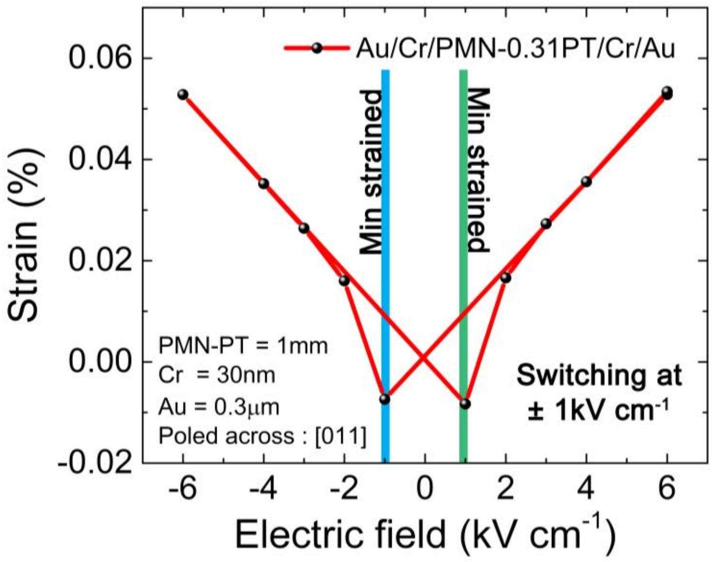

Supplementary figure S8: Quantification of strain over 1 mm PMNPT using impedance matching method. A strain switching is depicted on the applied electric field ~1 kV cm^{-1}. Strain ranging from 0.053% to -0.008% (or 0.53 to -0.08 μm) is induced in the 1 mm thick substrate with the application of an electric field in the range of ±6 kV cm^{-1}.

The dimension of the PMNPT crystal used for the measurement is 10.0*10.0*1.0 mm^3 (length*width*thickness). The impedance matching method[3] was used to measure the electric field-induced strain in the sample. This measurement was performed on one of the substrates drawn from the same batch which was used for the experiments. In this process, a thin film of Cr/Au (0.03/0.3 um) was sputtered on both sides of the substrate as electrodes. The sample was poled with different DC voltages along the [011] direction, and the piezoelectric constant, d33, was measured using Keysight E4490A Impedance Analyzer and an IAS ZJ-4AN meter under the conditions outlined by IEEE Standard on Piezoelectricity 176-1987. The poling conditions were direct current (DC) poling in the air with voltage ranging from -600 V to 0 V and 600 V to 0 V in

the steps of 100V. As observed in Supplementary figure S8, the minimum strain state is achieved near ±1 kV cm^{-1} (corresponding to ±100V), which is in close agreement with the THz-E hysteresis as shown in figure 1 of the manuscript (switching electric field ±1.2 kV cm^{-1}). A small discrepancy in the switching electric field is observed due to the use of a larger step size in the strain measurement. A strain ranging from 0.053% to -0.008% (or 0.53 to -0.08 µm) is induced in the 1 mm thick substrate with the application of an electric field in the range of ±6 kV cm^{-1}.

Supplementary Section S9: Calculation for THz electric field with ZnTe detector

We estimated the electric field strength of THz radiation. However, the setup yields an amplified voltage signal after passing through a pre-amplifier and a lock-in amplifier which is generally referred to in the arbitrary unit and is proportional to the actual THz electric field. To extract the field strength of THz radiation, one needs to estimate a THz-induced relative difference in the intensity of the *s*- and *p*-polarization of the probe laser as compared to its total intensity. Here, this relation between the intensities is dependent on the refractive index and electro-optic coefficient of the detector ZnTe crystal and is given as[4]

$$\frac{\Delta I}{I_0} = \frac{\omega\, n_0^3\, r_{41} E_{THz}\, d}{c} \qquad (S1)$$

where,

$$\Delta I = C.\Delta V \qquad (S2)$$

$$I_0 = C.V_{max}/2 \qquad (S3)$$

C is a constant, and ΔV is the difference in the measured voltage between the two photodiodes used for the balanced detection of the signal in the setup. ΔI is the balanced probe intensity between the *s*- and *p*-polarization of the probe laser. I_0 equals to the half of total probe intensity that can be measured in the absence of terahertz radiation. V_{max} is the measured voltage produced when total probe light is directed into a single photodiode. ω is the frequency of the laser, n_0= 3.22 [for 800nm light] is the refractive index of the electro-optic crystal[5]. r_{41}= 4.04 pm/V is the electro-optic coefficient for ZnTe[6] crystal. E_{THz} is the field strength of THz radiation, and d = 1 mm is the thickness of the ZnTe crystal. For an 800nm probe laser, the electric field strength of the THz radiation is given as

$$E_{THz} = \frac{\Delta V}{V_{max}} * 1.89\ * 10^4\ V/cm \qquad (S4)$$

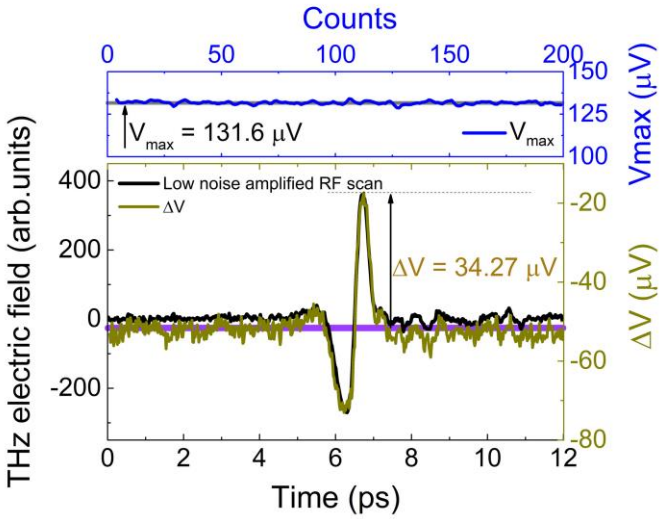

Supplementary figure S9: (Top) The measured voltage produced when total probe light is incident on a single photodiode (V_{max}), shown in a solid blue line. The measured signal is recorded in the absence of a THz electric field over 200 counts. **(Bottom)** The difference in the measured voltage between the two photodiodes is shown in the solid yellow line (ΔV) after the Wollaston prism splits the *s*- and *p*-polarization of the probe laser. Balanced photodetection with low noise trans-impedance gain of 175 x 10^3 V/A yields an amplified THz signal as shown in arbitrary units (solid black line).

To perform the measurement, as shown in Supplementary figure S9, a photodetector from Thorlabs, PDB210A/M, is used to yield individual photocurrents produced at each photodiode. The difference in signal from photodiodes was used to measure ΔV=34.27 µV. V_{max}=131.6 µV was measured when the entire probe laser was directed towards a single photodiode. Supplementary equation S4 was used to calculate the THz electric field amplitude (E_{THz}) equal to 4.92 kV cm^{-1}. In addition, PDB210A/M also performs a balanced photodetection with low noise trans-impedance gain of 175 x 10^3 V/A. Here, we measured an amplified THz signal with an amplitude of 365 mV, as shown by the solid black line in Supplementary figure S9. This amplified

electric field, when reported, is referred in arbitrary units to avoid any misconception. We then calculated a proportionality factor between the amplified signal and THz electric field; 1mV: 13.48 V/cm. All the measurements are performed in this low-noise configuration. Therefore, using the proportionality factor, all the THz arbitrary electric field was converted to kV cm^{-1}.

Supplementary Section S10: Modulation of electric field controlled THz amplitude

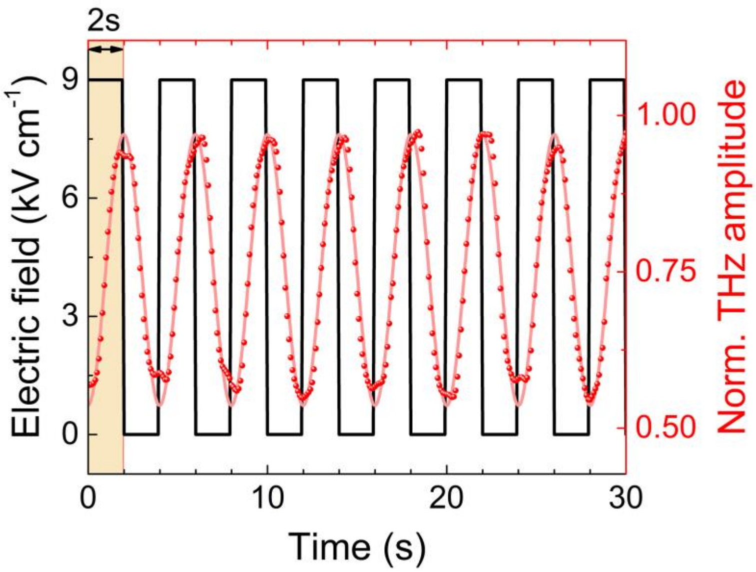

Supplementary figure S10: Electric field controlled THz amplitude modulation: A solid black line depicts the cycle of the applied electric field between 0 to 9 kV cm^{-1}. Red balls and a solid red line show the normalized THz amplitude modulation. THz amplitude is recorded in the steps of 100ms.

Supplementary figure S10 highlights the change of THz amplitude between two extreme states in a timescale of 2 seconds. Here, since PMNPT is a dielectric substrate with high resistivity, a rapid manifestation of a uniform electric field over a large area (1cm x 1cm x 0.1cm) becomes challenging and could not achieve a high modulation speed. However, to probe the ultrafast spin current using far-field terahertz radiation, a large-sized sample was used. The ultrafast spin current generation is an intrinsic phenomenon governed by the heterostructure properties and therefore provides a high device scalability down to micron[7] and sub-micron sizes. A recent work by Slawomir et al.[8] demonstrates strain application with frequency up to 100 MHz using micron-size

thin strips (6.7 μm x 90 μm x 500 μm) of ferromagnet/PMNPT. It is evident from the comparison with those micro-structures that our structure size is ~0.33 x 10^6 larger; hence downscaling our sizes is speculated to achieve a similar MHz modulation speed. An additional factor that plays an important role is the microstructure shape which endows a magnetic shape anisotropy[8], exhibiting a route to enhance the modulation speeds. Moreover, combined with careful control of electrode conductivity across the PMNPT substrate and laser fluence, much faster modulation speeds could be achieved.

References

1. Liang, W. *et al.* Anisotropic nonvolatile magnetization controlled by electric field in amorphous SmCo thin films grown on (011)-cut PMN-PT substrates. *Nanoscale* **11**, 246–257 (2018).
2. Nishikawa, H. *et al.* Rotation of the magnetic easy axis in La0.67Sr0.33MnO3 thin film on NdGaO3(112). *Appl. Phys. Lett.* **94**, 042502 (2009).
3. Rathod, V. T. A Review of Electric Impedance Matching Techniques for Piezoelectric Sensors, Actuators and Transducers. *Electronics* **8**, 169 (2019).
4. Yariv, A. & Yariv, A. *Optical electronics in modern communications*. (Oxford University Press, 1997).
5. Nahata, A., Weling, A. S. & Heinz, T. F. A wideband coherent terahertz spectroscopy system using optical rectification and electro-optic sampling. *Appl. Phys. Lett.* **69**, 2321–2323 (1996).
6. Turchinovich, D. Study of ultrafast polarization and carrier dynamics in semiconductor nanostructures a THz spectroscopy approach. (2004).
7. Olejník, K. *et al.* Terahertz electrical writing speed in an antiferromagnetic memory. *Science Advances* **4**, eaar3566 (2018).
8. Ziętek, S. *et al.* Electric-field tunable spin diode FMR in patterned PMN-PT/NiFe structures. *Appl. Phys. Lett.* **109**, 072406 (2016).
